# Using Lung Ultrasound Combined With N-terminal Pro-brain Natriuretic Peptide to Differentiate Acute Heart Failure From Chronic Obstructive Pulmonary Disease and Asthma in Emergency Department Patients Experiencing Acute Shortness of Breath

**DOI:** 10.7759/cureus.77171

**Published:** 2025-01-09

**Authors:** Ankit Sharma, Nidhi Kaeley, Nisarg Aravindan, Soumya Subhra Datta, Shivani Vasisht

**Affiliations:** 1 Department of Emergency Medicine, All India Institute of Medical Sciences, Rishikesh, Rishikesh, IND; 2 Department of Community and Family Medicine, St. John's Research Institute, Bengaluru, IND; 3 Department of Emergency Medicine, Rajarajeswari Medical College and Hospital, Bengaluru, IND; 4 Department of Dermatology, All India Institute of Medical Sciences, Rishikesh, Rishikesh, IND

**Keywords:** acute heart failure, acute shortness of breath, asthma, copd, emergency medicine, lung ultrasound point of care, nt-pro bnp

## Abstract

Introduction: Differentiating acute heart failure (AHF) from chronic obstructive pulmonary disease (COPD) or asthma is essential for prompt and appropriate treatment in patients presenting with acute shortness of breath in the emergency department (ED).

Aim: This study aimed to evaluate the diagnostic accuracy of bedside lung ultrasound, N-terminal pro-brain natriuretic peptide (NT-proBNP) level, and clinical criteria (using the modified Boston criteria) for differentiating AHF from COPD/asthma.

Materials and methods: This prospective cohort study was conducted in the Emergency Medicine Department of the All India Institute of Medical Sciences, Rishikesh, from June 2023 to December 2023. Patients presenting with acute shortness of breath were managed using clinical assessment (according to modified Boston criteria), lung ultrasound, and NT-proBNP measurements.

Results: Out of 104 patients, 45 were diagnosed with AHF, and 59 had pulmonary-related causes of shortness of breath. Lung ultrasound demonstrated a sensitivity of 100%, specificity of 62.3%, negative predictive value (NPV) of 100%, and positive predictive value (PPV) of 65.15% for diagnosing heart failure. NT-proBNP, with a cutoff value of 500 pg/mL, showed 100% sensitivity, 91.8% specificity, 100% NPV, and 89.58% PPV. The Boston modified criteria had a sensitivity of 76.74%, specificity of 88.52%, NPV of 84.38%, and PPV of 82.5%. A comparison of these three diagnostic methods revealed significant differences between the ultrasound findings and both NT-proBNP and modified Boston criteria (p < 0.05). The combination of ultrasound signs and NT-proBNP yielded 100% sensitivity, specificity, NPV, and PPV.

Conclusions: The integration of lung ultrasound, NT-proBNP level, and clinical criteria provides a reliable and rapid approach for differentiating AHF from COPD/asthma in the ED.

## Introduction

Shortness of breath is a “mismatch between central respiratory motor activity and incoming afferent information from receptors in the airways, lungs, and chest wall structures,” as per the American Thoracic Society. However, for patients, shortness of breath is a sensation of difficult or uncomfortable breathing. Various causes of shortness of breath are pulmonary (e.g., pneumothorax, pulmonary embolism, chronic obstructive pulmonary disease, COPD, asthma, aspiration, and pneumonia), cardiac (e.g., heart failure, myocardial ischemia or infarction, arrhythmias, valvular heart disease, and cardiac tamponade), metabolic (e.g., ketoacidosis, poisoning, and anemia), and others, including sepsis and psychogenic [1]. According to one report, the most common causes of shortness of breath in the emergency department (ED) were pneumonia (~25%), heart failure (~18%), COPD exacerbation (~15%), and asthma (~10%), with an overall inhospital mortality rate of 5%. Among all the causes, cardiac (heart failure, etc.) and respiratory (COPD and asthma) are the most common ones [2,3].

According to the European Society of Cardiology, heart failure is defined as a clinical syndrome characterized by typical symptoms (e.g., shortness of breath, ankle swelling, and fatigue) that may have signs (e.g., elevated jugular venous pressure, pulmonary crackles, and peripheral edema) caused by a structural and/or functional cardiac abnormality, leading to reduced cardiac output and/or elevated intracardiac pressures at rest or during stress. The annual mortality rate of heart failure is approximately 10%. It mainly occurs from sudden cardiac death (>50%) or organ dysfunction due to hypoperfusion [4]. In contrast, COPD is a heterogeneous lung condition characterized by chronic respiratory symptoms (dyspnea, cough, sputum production, and/or exacerbations) due to abnormalities of the airways (bronchitis and bronchiolitis) and/or alveoli (emphysema) that cause persistent, often progressive, airflow obstruction. Together, COPD and asthma have represented an extremely high social burden [5].

Thus, it is important to differentiate acute heart failure (AHF) from COPD and asthma to facilitate timely treatment. Clinical assessment plays an important role in differentiating the causes of acute shortness of breath, such as using the modified Boston criteria for heart failure, Global Initiative for Chronic Obstructive Lung Disease (GOLD) criteria for COPD, and asthma criteria [6-8]. However, it is not sufficient to detect the cause. Quick bedside tests, such as lung ultrasound and N-terminal pro-brain natriuretic peptide (NT-proBNP), are valuable tools for identifying the underlying cause of acute shortness of breath in the ED [6,9]. As per the Bedside Lung Ultrasound in Emergency protocol, a systematic approach to lung ultrasound is highly significant for differentiating AHF from COPD and asthma. A-lines on lung ultrasonography (USG) are horizontal artifacts arising from the pleural line generated by subpleural air, which blocks ultrasound waves. These A-lines with lung sliding can be present in patients with COPD/ asthma and in normal individuals. However, patients with AHF present with B-lines on lung USG. B-lines are hyperechoic, well-defined, comet-tail artifacts arising from the pleural line, spreading up indefinitely, erasing A-lines, and moving with lung sliding when lung sliding is present [10]. NT-proBNPs are cardiac hormones synthesized in response to mechanical stress and neurohormonal stimulation and secreted in the atria and ventricles. They help regulate electrolyte and water homeostasis, lipolysis, and blood pressure. The cutoff values of NT-proBNP for diagnosing heart failure vary across different age groups [11]. Usually, levels of more than 500 pg/mL point toward ventricular dysfunction [12].

Multiple previous studies have utilized point-of-care ultrasound and point-of-care cardiac markers, such as NT-proBNP and Troponin, and clinical criteria, such as modified Boston and Framingham criteria, individually to differentiate between cardiac and respiratory causes of shortness of breath. However, in this study, we assessed the combined diagnostic efficacy of bedside lung ultrasound, clinical criteria, and NT-proBNP levels. In a busy ED, it is difficult to differentiate between all life-saving causes of acute shortness of breath based on only one parameter. Thus, this study aims to evaluate the diagnostic accuracy of bedside lung ultrasound, NT-proBNP, and clinical parameters (according to the modified Boston criteria) for differentiating AHF-related acute shortness of breath from pulmonary (COPD/asthma)-related acute shortness of breath. Additionally, it seeks to assess the clinicodemographic, biochemical, and etiological profiles of patients presenting with acute shortness of breath to the ED, providing insights into an integrated approach for timely and accurate diagnosis in emergency care settings.

## Materials and methods

Study design and setting

This prospective cohort study was conducted to assess the diagnostic accuracy of bedside lung ultrasound, NT-proBNP, and clinical parameters for differentiating AHF-related acute shortness of breath from pulmonary-related acute shortness of breath. The study was carried out in the Emergency Medicine Department at the All India Institute of Medical Sciences (AIIMS), Rishikesh, over six months, from June 2023 to December 2023. The protocol received approval from the Institutional Ethics Committee (AIIMS/IEC/19/924). We calculated the sample size for this study, 104 patients, using Buderer’s statistical methodology [13]. All patients presenting with acute shortness of breath were triaged to the red area per the institutional protocol. Over the study period, 150 consecutive patients presenting with undifferentiated acute shortness of breath received emergency care, of which 104 patients were shortlisted based on the inclusion criteria. This was in accordance with the sample size calculated for the study, and informed written consent was obtained from all patients for their participation. Following emergency care, all patients were admitted to the emergency intensive care unit either for clinical reasons or to meet the study’s inclusion criteria and were monitored until discharge.

Study population

The study included patients who presented with acute shortness of breath as their primary complaint, either sudden onset of shortness of breath without a history of chronic shortness of breath or an exacerbation of chronic shortness of breath. Individuals under 18 years of age were excluded. Additionally, patients with trauma, chronic kidney disease, and acute coronary syndrome, unless their primary complaint is shortness of breath, were excluded. Individuals suffering from respiratory conditions other than COPD or asthma, such as pneumonia, pulmonary embolism, carcinoma, pneumothorax, pleural effusion, drug intoxication, anaphylactic reactions, upper airway obstruction, bronchial stenosis, and gastroesophageal reflux disorder, were also excluded.

Data collection

A detailed history, including their past medical records, was obtained from the patients, and findings were noted. A thorough clinical examination was performed on all patients, and findings were noted on a prefilled Google form. Modified Boston criteria were applied for all patients, and patients were subdivided for heart failure as definite (score 8-12 points), possible (score 5-7 points), and unlikely (score 4 or less points) [6].

Electrocardiogram, arterial blood gas analysis, and cardiac markers, such as troponin I, were performed in all patients. D-dimer was also administered according to the needs of the patient. Bedside echocardiography was performed in all patients. To further evaluate patients with suspected obstructive causes of shortness of breath, we incorporated the criteria for assessing severe asthma and exacerbations of chronic COPD while maintaining a modified Boston criteria threshold for heart failure of ≤ 5 [7,8].

The final hospital diagnosis of acute shortness of breath related to heart failure and pulmonary-related cause was based on established criteria consistent with the traditional instruments, such as chest X-ray, echocardiographic examination, cardiac functional assessment (exercise test), pulmonary function test, complete blood count, biochemistry, and, when necessary, invasive investigation or angiography [14-17].

During the initial assessment, before administering any medications, a 5-mL blood sample was obtained and collected in a tube containing edetate calcium disodium for NT-proBNP. The NT-proBNP test was performed in the ED itself and completed within 15 minutes [6,18].

Simultaneously, the bedside thoracic ultrasound was conducted following the protocol outlined by Cardinale et al., Volpicelli et al., and Liteplo et al., involving the scanning of eight zones of the lungs (two anterior and two lateral zones on each side of the thorax) using a portable ultrasound machine manufactured by SonoSite (FUJIFILM Sonosite, Inc., Bothell, WA) [9,19,20]. Five experienced emergency physicians were involved in the investigations, and they were tasked with identifying the presence or absence of three or more B-lines in each of the eight zones within one minute. B-lines, also known as comet-tail signs, are hyperechoic reverberation artifacts originating at the pleural line that extends vertically to the bottom of the screen. A positive ultrasound examination, as defined by Cardinale et al. and Volpicelli et al., requires two or more positive zones bilaterally out of the eight zones measured [9,19].

NT-proBNP measurements and ultrasound examinations were conducted immediately upon the patient's arrival at the ED before administering any medication. This approach ensured that the results were not influenced by treatment. The individuals responsible for diagnosing the patients (emergency physicians, internists, cardiologists, and/or intensive care physicians upon hospital discharge, who determined the final diagnosis) were unaware of the NT-proBNP results. Moreover, the investigators responsible for the NT-proBNP analysis did not contribute to the final diagnosis. However, emergency physicians were aware of the ultrasound findings because bedside lung ultrasound is a routine assessment method in our ED. To mitigate bias, emergency physicians recorded the ultrasound findings without allowing them to impact the diagnosis. Furthermore, the individuals making the diagnosis in the hospital were blinded to the ultrasound findings.

**Figure 1:**
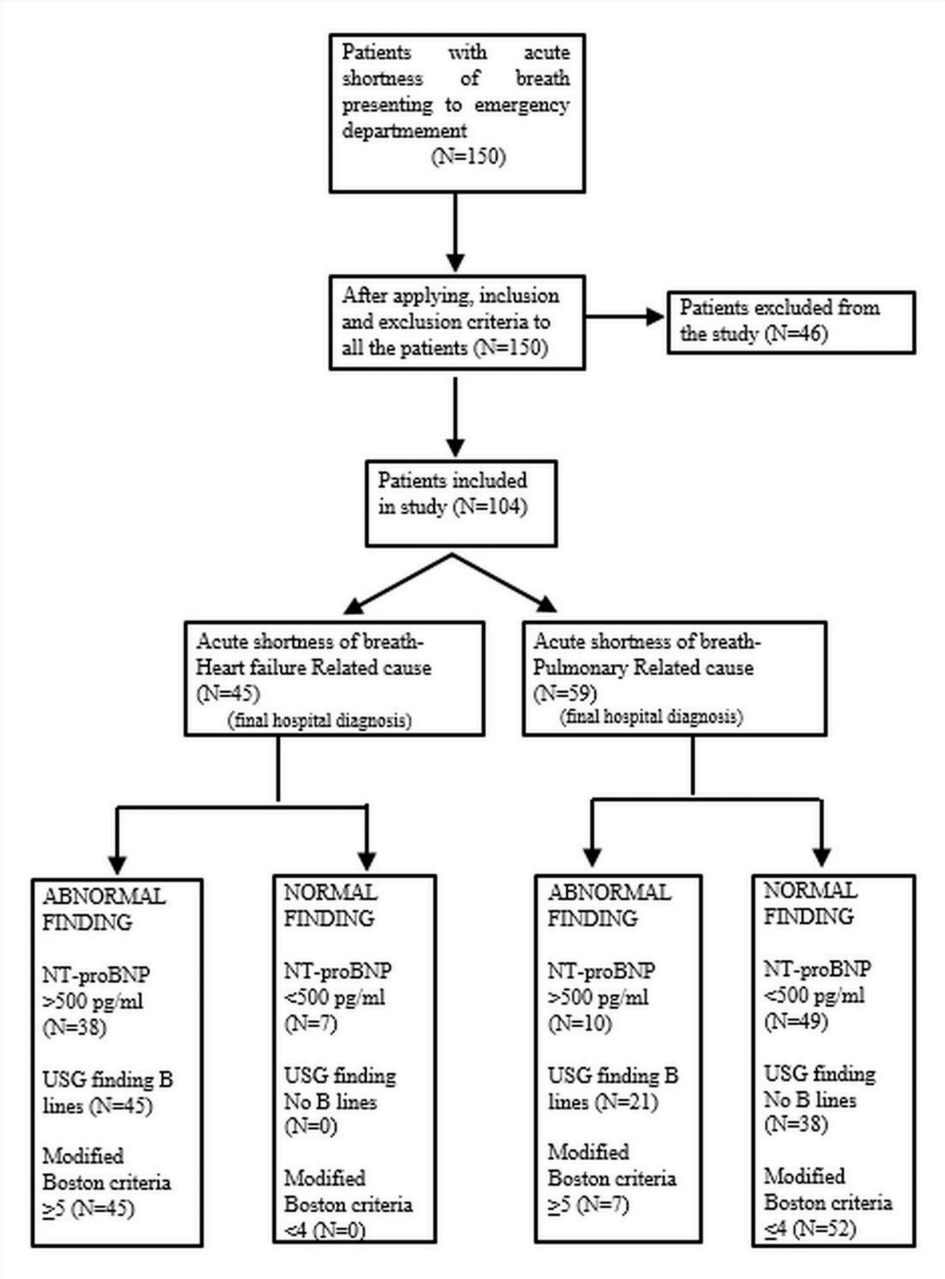
Study flowchart NT-proBNP: N-terminal pro-brain natriuretic peptide; USG: ultrasonography

The faculty emergency medicine/cardiologist/pulmonologist confirmed the final diagnosis, using standard diagnostic criteria with the help of echocardiography, pulmonary function tests, and other biochemical and hematological tests. All emergency physicians who participated in the study underwent a standard point-of-care USG course, and the emergency medicine faculty confirmed the findings. The study flowchart is given in Figure 1.

Statistical analysis

Univariate comparisons utilized the χ^2^ test/Fisher's exact test for categorical variables and an unpaired t-test for continuous variables with a normal distribution, including age, pulse rate, partial pressure of end-tidal carbon dioxide, NT-proBNP, arterial oxygen saturation, and modified Boston criteria for heart failure. Sensitivity, specificity, negative predictive value (NPV), positive predictive value (PPV), positive likelihood ratio (LR+), and negative likelihood ratio (LR-) were estimated for clinical assessment (based on the modified Boston criteria), NT-proBNP, ultrasound examination, and a combination of ultrasound and NT-proBNP. Comparison among these four methods was conducted using the χ^2^ test with Bonferroni correction for multiple comparisons. Additionally, the area under the receiver operating characteristic (AUROC) curve was used to evaluate the diagnostic accuracy of the four methods in distinguishing heart failure-related acute shortness of breath from pulmonary-related acute shortness of breath. Single areas were calculated and compared using univariate Z-score testing. The technique proposed by Hanley and McNeil and Januzzi et al. was employed to compare the areas under different curves. Statistical analyses were performed using the SPSS software (SPSS Inc., Chicago, IL), and AUROC analysis was conducted using the Analyse-It software (Analyse-it Software, Ltd., Leeds, UK) [21,22].

## Results

During the study period, the emergency team treated 150 patients with acute shortness of breath; however, only 45 patients with heart failure-related acute shortness of breath and 59 patients with pulmonary-related acute shortness of breath were included in this study. After applying the inclusion and exclusion criteria, forty-six patients were excluded from the study. The clinical and demographic characteristics of the patients are summarized in Tables 1-4.

**Table 1:** Univariate descriptive statistical analysis for all clinicodemographic categorical variables (history-based) relevant to diagnosing acute shortness of breath due to heart failure or pulmonary cause (n = 104) ^*^Significant pairwise difference mMRC: modified Medical Research Council; DM2: diabetes mellitus type 2; COPD: chronic obstructive pulmonary disease; AHF: acute heart failure

Clinicodemographic categorical variables		Final diagnosis, n (%)		Chi-square	p value
		Pulmonary-related dyspnea	AHF-related dyspnea		
Sex	Female	22 (37.3%)	20 (44.4%)	0.543	0.461
	Male	37 (62.7%)	25 (55.6%)	-	-
Shortness of breath mMRC grade	II	6 (10.2%)	1 (2.2%)	3.419	0.181
	III	11 (18.6%)	6 (13.3%)	-	-
	IV	42 (71.2%)	38 (84.4%)	-	-
Nocturnal shortness of breath	Absent	50 (84.7%)	15 (33.3%)	28.791	<0.001^*^
	Present	9 (15.3%)	30 (66.7%)^*^	-	-
Orthopnea	Absent	44 (74.6%)	10 (22.2%)	28.031	<0.001^*^
	Present	15 (25.4%)	35 (77.8%)^*^	-	-
Cough	Absent	10 (16.9%)	8 (17.8%)	0.012	0.912
	Present	49 (83.1%)	37 (82.2%)	-	-
Associated expectoration with cough	Absent	19 (32.2%)	20 (44.4%)	1.632	0.201
	Present	40 (67.8%)	25 (55.6%)	-	-
History of fever	Absent	19 (32.2%)	33 (73.3%)^*^	17.275	<0.001^*^
	Present	40 (67.8%)^*^	12 (26.7%)	-	-
History of asthma	No	54 (91.5%)	43 (95.6%)	0.660	0.416
	Yes	5 (8.5%)	2 (4.4%)	-	-
Past history of COPD	No	24 (40.7%)	37 (82.2%)^*^	18.1	<0.001^*^
	Yes	35 (59.3%)	8 (17.8%)	-	-
Past history of DM2	No	45 (76.3%)	18 (40.0%)	14.064	<0.001^*^
	Yes	14 (23.7%)	27 (60.0%)^*^	-	-

**Table 2:** Univariate descriptive statistical analysis for all clinicodemographic categorical variables (history and examination-based) relevant to diagnosing acute shortness of breath due to heart failure or pulmonary cause (n = 104) ^*^Significant pairwise difference AHF: acute heart failure; HTN: hypertension; TB; tuberculosis; JVP: jugular venous pressure

Clinicodemographic categorical variables		Final diagnosis, n (%)		Chi-square	p value
		Pulmonary-related dyspnea	AHF-related dyspnea		
Past history of HTN	No	45 (76.3%)	19 (42.2%)	12.504	<0.001^*^
	Yes	14 (23.7%)	26 (57.8%)^*^	-	-
Past history of pulmonary TB	No	47 (79.7%)	42 (93.3%)^*^	3.866	0.055^*^
	Yes	12 (20.3%)	3 (6.7%)	-	-
Past history of ischemic heart disease	No	57 (96.6%)	30 (66.7%)	16.739	<0.001^*^
	Yes	2 (3.4%)	15 (33.3%)^*^	-	-
Past history of arrhythmias	No	57 (96.6%)	38 (84.4%)	4.780	0.038^*^
	Yes	2 (3.4%)	7 (15.6%)^*^	-	-
Past history of heart failure	No	56 (94.9%)	24 (53.3%)	24.866	<0.001^*^
	Yes	3 (5.1%)	21 (46.7%)^*^	-	-
Heart rate	<60	0 (0.0%)	2 (4.4%)	6.903	.075
	>150	0 (0.0%)	2 (4.4%)	-	-
	100-150	23 (39.0%)	21 (46.7%)	-	-
	60-100	36 (61.0%)	20 (44.4%)	-	-
Systolic blood pressure	<140	38 (64.4%)	24 (53.3%)	2.432	0.488
	>180	1 (1.7%)	0 (0.0%)	-	-
	140-159	16 (27.1%)	16 (35.6%)	-	-
	160-179	4 (6.8%)	5 (11.1%)	-	-
Elevated JVP	No	55 (93.2%)	12 (26.7%)	49.33	<0.001^*^
	Yes	4 (6.8%)	33 (73.3%)^*^	-	-
Pedal edema	Absent	49 (83.1%)	8 (17.8%)	43.913	<0.001^*^
	Present	10 (16.9%)	37 (82.2%)^*^	-	-
Wheeze on auscultation	Absent	15 (25.4%)	35 (77.8%)^*^	28.031	<0.001^*^
	Present	44 (74.6%)	10 (22.2%)	-	-

**Table 3:** Univariate descriptive statistical analysis for all clinicodemographic categorical variables (based on clinical and laboratory findings) relevant to diagnosing acute shortness of breath due to heart failure or pulmonary cause (n = 104) ^*^Significant pairwise difference FiO_2_: fraction of inspired oxygen; PaO_2_: partial pressure of oxygen in arterial blood; COPD: chronic obstructive pulmonary disease; NT-proBNP: N-terminal pro-brain natriuretic peptide; ECG: electrocardiogram; MI: myocardial infarction; LV: left ventricular; LVH: left ventricular hypertrophy; RAD: right axis deviation; LBBB: left bundle branch block; AHF: acute heart failure

Clinicodemographic categorical variables		Final diagnosis, n (%)		Chi-square	p value
		Pulmonary-related dyspnea	AHF-related dyspnea		
Crepitations	Absent	31 (52.5%)	1 (2.2%)	30.34	<0.001^*^
	Present	28 (47.5%)	44 (97.8%)^*^	-	-
Murmurs	Absent	58 (98.3%)	39 (86.7%)	5.508	<0.019
	Present	1 (1.7%)	6 (13.3%)	-	-
ECG findings	Atrial flutter/fibrillation	1 (1.7%)	7 (15.6%)^*^	35.57	<0.001^*^
	Changes suggestive of COPD	7 (11.9%)	2 (4.4%)	-	-
	LVH, RAD, poor R wave progression	0 (0.0%)	1 (2.2%)	-	-
	Old LBBB	0 (0.0%)	1 (2.2%)	-	-
	Sinus rhythm	51 (86.4%)	20 (44.4%)	-	-
	ST-T changes suggestive of recent/remote MI	0 (0.0%)	14 (31.1%)	-	-
Troponin I >0.05 ng/mL	No	58 (98.3%)	40 (88.9%)	4.16	0.083
	Yes	1 (1.7%)	5 (11.1%)	-	-
PaO_2_/FiO_2_ ratio	100-200	13 (22.0%)	9 (20.0%)	1.46	0.691
	200-300	20 (33.9%)	13 (28.9%)	-	-
	300 or more	23 (39.0%)	18 (40.0%)	-	-
	Less than 100	3 (5.1%)	5 (11.1%)	-	-
Lung ultrasound	A-lines	38 (64.4%)	0 (0.0%)	45.67	<0.001^*^
	B-lines	21 (35.6%)	45 (100.0%)	-	-
LV ejection fraction	<40%	2 (3.4%)	33 (73.3%)^*^	58.01	<0.001^*^
	>50%	40 (67.8%)	5 (11.1%)	-	-
	40%-50%	17 (28.8%)	7 (15.6%)	-	-
Modified Boston criteria score for diagnosing heart failure	Definite (score 8-12)	1 (1.7%)	39 (86.7%)^*^	87.807	<0.001^*^
	Possible (score 5-7)	6 (10.2%)	6 (13.3%)	-	-
	Unlikely (score 4 or less)	52 (88.1%)	0 (0.0%)	-	-
NT-ProBNP	<500	49 (83.1%)^*^	7 (15.6%)	46.797	<0.001^*^
	≥500	10 (16.9%)	38 (84.4%)^*^	-	-

**Table 4:** Univariate descriptive statistical analysis for all clinicodemographic continuous variables relevant to diagnosing acute shortness of breath due to heart failure or pulmonary cause (n = 104) ^*^Significant pairwise difference. CI: confidence interval; pCO_2_: partial pressure of carbon dioxide; NT-ProBNP: N-terminal pro-brain natriuretic peptide; CBC: complete blood count; TLC: total leukocyte count; AHF: acute heart failure; SGOT: serum glutamic oxaloacetic transaminase; SGPT: serum glutamate pyruvate transaminase; GGT: gamma-glutamyl transferase

Clinicodemographic continuous variables	Final diagnosis				T test mean difference	95% CI		P value
	Pulmonary-related dyspnea		AHF-related dyspnea					
	Mean	Standard deviation	Mean	Standard deviation		Lower	Upper	
Patient age	55	17	54	16	1.588	-4.845	8.020	0.626
Arterial pCO_2_	56.9	22.0	41.1	12.1	15.8007	9.0811	22.5202	<0.001^*^
Mean NT-ProBNP level	289	215	677	221	-388.149	-473.883	-302.416	<0.001^*^
Mean arterial O_2_ saturation (%)	73	17	8	16	-2.159	-8.804	4.487	0.521
D-dimer level	499	909	408	296	91.540	-188.682	371.763	0.518
Hemoglobin CBC	12.40	2.52	11.66	2.57	0.73719	-0.26068	1.73507	0.146
Hematocrit %	38.7	8.4	37.7	8.6	1.0224	-2.3077	4.3525	0.544
TLC	13,925	4,580	13,154	5,368	771.525	-1,165.950	2,709.000	0.431
Neutrophils %	65.81	19.59	66.78	17.71	-0.96755	-8.34928	6.41417	0.795
Lymphocytes %	26.14	13.76	22.83	11.77	3.30382	-1.77526	8.38289	0.200
Platelets	203,063	81,269	236,953	89,886	-33,890.621	-67,811.349	30.106	<0.047^*^
Blood urea level	40	14	47	27	-6.552	-14.599	1.495	0.139
Serum creatinine level	1.11	0.45	2.26	3.24	-1.14840	-2.12918	-0.16762	<0.023^*^
Total bilirubin	1.22	0.50	1.66	1.04	-0.43818	-0.77505	-0.10131	<0.012^*^
Direct bilirubin	0.62	0.30	0.79	0.48	-0.16834	-0.32997	-0.00671	<0.030^*^
SGOT	71	91	103	153	-32.252	-79.869	15.365	0.213
SGPT	80	133	127	212	-46.542	-114.046	20.963	0.202
GGT	115	63	116	65	-0.902	-26.049	24.244	0.943
Alkaline phosphatases	126	61	134	60	-7.548	-31.331	16.235	0.530

Patients with AHF were of comparable age (mean ages 54± 16 years) as those with pulmonary-related causes of acute shortness of breath (mean ages 55 ± 17 years). No significant differences exist in the patients' sex at the final diagnosis. The presence of nocturnal dyspnea, orthopnea, pedal edema, and elevated jugular venous pressure was significantly higher in patients with AHF. In contrast, fever was significantly higher in patients with COPD and asthma. History of diabetes mellitus type 2 and hypertension was higher in patients with AHF, whereas history of COPD was higher in patients with pulmonary-related dyspnea.

The feasibility of ultrasound examination in the emergency setting was 100%, with examination consistently taking less than one minute. The sensitivity, specificity, PPV, NPV, LR+, LR-, and AUROC curve values for ultrasound examinations (cutoff point: two or more positive zones bilaterally), modified Boston criteria (cutoff point: total 5 points), NT-proBNP (cutoff point: 500 pg/mL), and a combination of ultrasound examination with NT-proBNP, is mentioned in Table 5.

**Table 5:** Vital indicators of ultrasound examination accuracy, modified Boston examination, NT-proBNP level, and combination of ultrasound examination and NT-proBNP level CI: confidence interval; NT-proBNP: N-terminal pro-brain natriuretic peptide; NPV: negative predictive value; PPV: positive predictive value; AUROC: area under receiver operating characteristic

Characteristic	Ultrasound examination (95% CI)	Modified Boston criteria scoring (95% CI)	NT-proBNP (95% CI)	Ultrasound examination + NT-proBNP (95% CI)	P value
Sensitivity	100% (91.8-100)	76.74% (62.26-86.85)	100% (91.8-100)	100% (92.59-100)	<0.001^*^
Specificity	62.3% (49.75-73.39)	88.52% (78.16-94.33)	91.8% (82.21-96.45)	100% (93.58-100)	<0.001^*^
PPV	65.15% (53.11-75.52)	82.5% (68.05-91.25)	89.58% (77.83-95.47)	100% (92.59-100)	<0.001^*^
NPV	100% (90.82-100)	84.38% (73.57-91.29)	100% (93.58-100)	100% (93.58-100)	<0.001^*^
Likelihood ratio positive	2.652 (2.436-2.888)	6.688 (4.964-9.009)	12.2 (8.244-18.06)	Infinite	<0.001^*^
Likelihood ratio negative	0	0.2627 (0.2149-0.3211)	0	0	<0.001^*^
AUROC	0.81 (0.729-0.89)	0.826 (0.739-0.894)	0.959 (0.918-1)	0.99 (0.98-1)	<0.001^*^

Upon comparing the methods, significant differences were found between ultrasound signs and NT-proBNP (p < 0.05) and between ultrasound signs and modified Boston criteria (p < 0.05). All 10 patients with false-positive results using the NT-proBNP method had values higher than 500 pg/mL and a history of heart failure. In these cases, the absence of comet-tail signs excluded heart failure in patients with pulmonary-related dyspnea. Conversely, 21 patients with false-positive results using the ultrasound method had NT-proBNP levels less than 500 pg/mL and a history of COPD/asthma. Thus, using the NT-proBNP value, heart failure can be excluded in ultrasound-positive patients with pulmonary-related dyspnea.

## Discussion

Acute shortness of breath accounts for up to 5% of ED presentations, approximately 10% of ward admissions, and 20% of intensive care unit admissions [4]. This condition brings the need for study on acute shortness of breath.

Early detection of cause and intervention in all cases of acute shortness of breath can save many lives. This made it necessary for the need for quick bedside investigations in the form of lung ultrasound and NT-proBNP in the ED to differentiate the causes of acute shortness of breath as heart failure and COPD/ asthma. Previous studies have established the utility of lung ultrasound in diagnosing heart failure. For instance, Pivetta et al. reported a sensitivity of 97% and a specificity of 92% for lung ultrasound in diagnosing AHF in the ED [23]. Our findings of a sensitivity of 100% and specificity of 62.3% are consistent with the high sensitivity reported in the literature but show a lower specificity. This discrepancy could be due to differences in study populations or operator expertise. Unlike Pivetta et al., who included a broader patient population, our study specifically focused on differentiating heart failure from COPD and asthma, which may have contributed to the variability in specificity.

NT-proBNP is another quick and well-established marker used to diagnose heart failure. It is a biologically inactive peptide secreted from cardiomyocytes under stressful conditions [11]. Among all the patients with heart failure-related dyspnea in our study, 84.4% had NT-proBNP levels of more than 500 pg/mL. The diagnostic performance of NT-proBNP in our study was in agreement with findings from previous studies. Mueller et al. found that NT-proBNP levels had an area under the curve (AUC) of 0.91 in distinguishing heart failure from other causes of shortness of breath in the ED [24]. Our study's AUC of 0.959 for NT-proBNP indicates a similarly high diagnostic accuracy, reinforcing the biomarker's robustness in clinical practice. The slightly higher AUC in our study might be attributed to advancements in assay techniques or differences in patient cohorts, such as excluding nonpulmonary causes (other than COPD/Asthma) of shortness of breath.

In our study, ultrasound examination was the most effective standalone diagnostic tool for confirming AHF in the ED. Compared with clinical assessment employing modified Boston criteria and NT-proBNP testing, lung ultrasound exhibited notably superior diagnostic accuracy, as evidenced by its significantly higher AUROC. However, the combination of ultrasound examination and NT-proBNP testing demonstrated higher reliability in identifying AHF and distinguishing it from COPD/Asthma. The amalgamation of these two methods demonstrated outstanding statistical significance: 100% sensitivity, specificity, negative predictive value (NPV), positive predictive value (PPV), 99% AUROC curve, infinite LR+, and zero LR-. The combined use of lung ultrasound and NT-proBNP has been explored in previous studies with varying degrees of success. Volpicelli et al. demonstrated that combining lung ultrasound with clinical assessment improved the diagnostic accuracy of heart failure [19]. Our study further extended these findings by quantifying the added value of NT-proBNP, resulting in a combined AUC of 0.99. This significant improvement underscores the synergistic effect of integrating imaging and biomarker data, a conclusion supported by studies like those by Platz et al., who reported improved diagnostic accuracy with combined modalities in patients with acute shortness of breath [25]. Prosen et al. also reported similar findings in their study, but they were conducted in a prehospital setting [26]. However, the availability of equipment, the accuracy of operation, the interpretation of USG, and the performance of NT-proBNP by prehospital staff are always challenging tasks in developing countries. All these can be ensured by emergency physicians in EDs in developing countries. Liteplo et al. conducted a study in which the combination of detecting B-lines on ultrasound and elevated NT-ProBNP levels was found to be highly indicative of AHF as the cause of shortness of breath, which is equivocal to our study [20].

If all patients with acute shortness of breath undergo lung USG in the emergency, physicians can diagnose COPD/asthma in no time as those patients will have no B-lines on USG, and those with B-lines, NT-proBNP, should be measured. High NT-proBNP levels will help in the diagnosis of heart failure. Early detection of the cause of acute shortness of breath and appropriate intervention can save many lives. Further detailed evaluation can be performed for all patients after admission to the intensive care units and wards of the hospital.

Limitations

We included only patients with heart failure and COPD/asthma in our statistical analysis. In our study, we did not consider patients with other causes of acute shortness of breath, which limited our knowledge of lung USG and NT-proBNP in other causes of acute shortness of breath. Although our study provides robust evidence supporting the combined use of lung ultrasound and NT-proBNP, it is important to recognize its limitations and the need for future research. Also, our results lack a correlation between the degree of shortness of breath and intervention. The single-center design may limit generalizability, and operator-dependent lung ultrasound remains challenging. Future multicenter studies should aim to validate these findings across diverse clinical settings and explore standardized training protocols to minimize operator variability. Furthermore, the influence of comorbidities on NT-proBNP levels requires careful interpretation. Research focusing on refining cutoff values for different patient subgroups could enhance the specificity of NT-proBNP. In addition, integrating other novel biomarkers or advanced imaging techniques could further improve diagnostic accuracy and patient care for acute shortness of breath.

## Conclusions

The study demonstrates that the combined use of lung ultrasound and NT-proBNP offers an exceptionally reliable and rapid diagnostic approach for differentiating AHF from COPD and asthma in emergency settings. This combination achieves 100% sensitivity, specificity, and predictive accuracy, significantly enhancing diagnostic precision over individual methods. Integrating these modalities into routine emergency care can facilitate timely interventions and improve patient outcomes in cases of acute shortness of breath.
